# An Influence of Actuator Gluing on Elastic Wave Excited in the Structure

**DOI:** 10.3390/ma17092160

**Published:** 2024-05-06

**Authors:** Dominika Ziaja, Michał Jurek

**Affiliations:** Department of Structural Mechanics, Rzeszow University of Technology, ul. Poznańska 2, 35-084 Rzeszów, Poland; mjurek@prz.edu.pl

**Keywords:** guided waves, laser Doppler vibrometry, genetic algorithms

## Abstract

In this article, the practical issues connected with guided wave measurement are studied: (1) the influence of gluing of PZT plate actuators (NAC2013) on generated elastic wave propagation, (2) the repeatability of PZT transducers attachment, and (3) the assessment of the possibility of comparing the results of Laser Doppler Vibrometry (LDV) measurement performed on different 2D samples. The consideration of these questions is crucial in the context of the assessment of the possibility of the application of the guided wave phenomenon to structural health-monitoring systems, e.g., in civil engineering. In the examination, laboratory tests on the web of steel I-section specimens were conducted. The size and shape of the specimens were developed in such a way that they were similar to the elements typically used in civil engineering structures. It was proved that the highest amplitude of the generated wave was obtained when the exciters were glued using wax. The repeatability and durability of this connection type were the weakest. Due to this reason, it was not suitable for practical use outside the laboratory. The permanent glue application gave a stable connection between the exciter and the specimen, but the generated signal had the lowest amplitude. In the paper, the new procedure dedicated to objective analysis and comparison of the elastic waves propagating on the surface of different specimens was proposed. In this procedure, the genetic algorithms help with the determination of a new coordinate system, in which the assessment of the quality of wave propagation in different directions is possible.

## 1. Introduction

Guided wave propagation is one of the mechanical phenomena used in the damage detection of engineering structures [1,2]. Particles of a solid continuous medium, thrown out of equilibrium, vibrate. The excited waves, traveling throughout the material, reflect from boundaries or defects in the material. It does not destroy the element. For this reason, the analysis of wave propagation is a valuable source of information about the structure of the material, as well as the condition of the examined specimen.

The NDT techniques, connected with guided wave measurements, can be divided into (1) contact-less and (2) contact methods. In the first case, Laser Doppler Vibrometry (LDV) is used, while in the second one, wave measurement can be made using ultrasonic probes, piezoelectric wafers and piezocomposite transducers, inter-digital transducers, or fiber-optic sensors [1]. Regardless of the selected wave measurement method, the wave has to be excited in the material. It also can be made contact-less (e.g., using air-coupled excitation or laser sources [3]) or using exciters touching to the examined surface [4]. As the most popular exciters, PZT wafers/elements can be pointed. They are glued on the surface of the structure or inside it (e.g., between layers of composites [5] or embedded inside the specimen on a reinforcement bar [6]). The last case is possible only if SHM system installation was planned before the production of the structure. In many cases, the decision to structure monitoring is made after that, so this solution is not possible. In the case of real structures operating in real conditions, the problem is the inability to ensure stable and repeatable excitation conditions using the air-coupled method. The influence of the external environment can significantly affect the excited wave. Due to these reasons, it seems like the gluing of exciters on the structure surface gives the greatest scope of applications for SHM [7,8]. However, as shown in this article, the case of gluing exciters influences excited wave propagation.

The guided waves excited using PZT sensors were applied in defect detection in welded composite joints [9], estimation of the depth of cracks in thick steel beams [10] or plates [11], or de-bonding detection between concrete block and CFRP reinforcement [12]. This measurement technique was also used to monitor concrete curing [7] and corrosion identification [13]. Surface-mounted PZT exciters were used in delamination detection in thick composite laminates [14] and debonding detection between the steel deck and ultra-high performance concrete layer of a lightweight composite bridge [15].

The influence of the thickness of the bonding layer between PZT transducers and host structures was studied by Kaur and Bahalla in [16,17]. They tried to use the PZT transducers for energy harvesting and showed that the higher the thickness of the bonding and the lower the shear modulus of the bond layer, the smaller the amount of obtained energy. They did not observe the inverse piezoelectric effect (the guided wave was not excited, but it can be supposed that the bonding layer also influences the vibration generated by PZT transducers to the host structures).

One more thing should be noted, namely, if the gluing of sensors is important, can the exciters be glued twice in the same way? This question is significant in SMH system configurations. It is possible that during the exploration of the structure, the exciter will be broken and require to be exchanged [8]. Will the new exciter (even in the same size, type, and excitation signal) generate the same wave in the structure? Tracking the examples in the literature can show, that there is a lack of direct answers. In many articles, individual solutions are presented [11,13,18,19,20,21]. Despite how interesting they are, they do not raise issues of sensor attachment influence on excited and registered signals. In the articles on the use of elastic waves [12,22], the authors take into account diverse sets of samples and the influence of sample characteristics on the wave parameters, but the issue of the influence of bonding on the excited wave is not discussed or considered. Potential discrepancies between the analyzed signals can be results from gluing the sensors, which was mentioned in [22], or the specimen surface roughness [7]. So, simply comparing the signals obtained during examination on different specimens, without taking into account the influence of the sensors sticking, can be risky.

Another problem connected with sensors attaching is the repeatability of measurement on different specimens [22]. The development of appropriate procedures for the comparison of excited signals in many specimens is important from a practical point of view. Due to the sensor gluing, the real excited wave can be different from the generated one.The main purpose of this article is to show that the problems with the repeatable sticking of the PZT transducers exist and need to be taken into account before applying the guided wave measurement to the SHM of real structures.

In this article, such issues are also discussed: (1) qualitative and quantitative comparison of measurements made in different areas, and (2) comparison of the influence of different glues on wave excited with surface-stuck PZT.

## 2. Material and Methods

The examinations were conducted on steel specimens. The shape of the specimens resulted from their similarity to the parts of real, engineering steel structures in the range of the shape as well as the dimension. Under observation were the parts of the webs of the I-section beams (despite their vertical location on the measuring stand). Each of the beams was made with IPE300, so the thickness of the web should be 7.1 mm with 1 mm accuracy. The material properties were experimentally determined using ultrasound techniques (Young’s modulus E = 216.65 GPa, Poisson’s coefficient ν= 0.285, and shear modulus G = 84.28 GPa).

**Figure 1:**
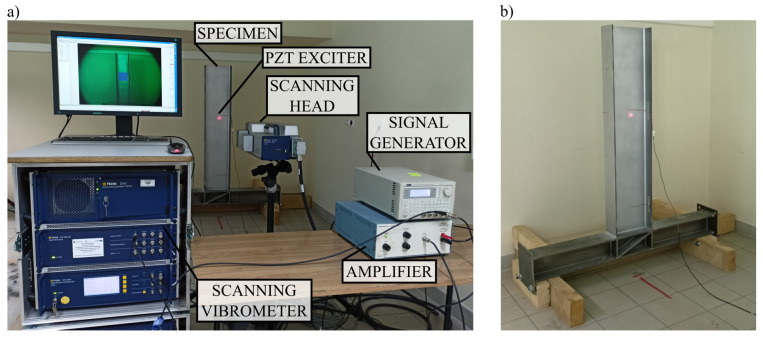
(**a**) Measuring stand and (**b**) the specimen under examination.

The elastic wave propagation observation was made by a non-contact measurement technique, which was Laser Doppler Vibrometry (LDV). This technique allows measurements in a very dense mesh of measuring points. Thanks to that, the visualization of propagating waves is possible. The measuring stand is shown in Figure 1. It consists of the equipment for wave excitation: the signal generator (TTi Thurlby Thandar Instruments typu TG1010i Function Generator, Huntingdon, UK), amplifier (Piezo Systems Inc. EPA-104, Cambridge, MA, USA), PZT sensors, and, for wave registration, Polytec Scanning Vibrometer PSV-400-3D (Waldbronn, Germany). Due to the measurement being bounded to a direction perpendicular to the surface of specimens, only one scanning head was used.

The real dimension of the observed area (measured on the specimen surface) was 205 mm width and 200 mm high, and there were 5183 measuring points, arranged regularly. The PZT exciter was glued approximately in the middle of the area.

The wave excitations were made using plate actuators NAC2013 (dimensions: 5 × 5 × 2 mm) and PZT actuators by Noliac (www.noliac.com/products/actuators/plate-actuators/show/nac2013, accessed on 10 January 2024). During measurements, the influence of the gluing method was analyzed, so five different types of glue were tested as shown in Table 1. In this table, also the advantages and disadvantages of the application of each of them are formulated based on long-term, personal experiences in the field of elastic wave measurements experiments. The identified advantages and disadvantages result from the different chemical compositions of individual adhesives, which translate into features such as setting speed, strength, the flexibility and durability of the connection, and resistance to accidental loads. Moreover, the chemical composition of the adhesive and the mechanical properties of the connection are related to the ability to transfer the excited wave from the PZT to the analyzed element.

Two types of excitation were tested, namely, the 2.5 and 3.5 sine waves, both cases modulated with the Hanning window. The frequency of the generated signal was different for each of them, with the aim to obtain the same operational frequency 100 kHz, and they were, respectively, 40 kHz and 28.571 kHz. The change in the type of excitation signal, with the same value of operational frequency and amplitude, influences the energy of excitation. In analyzed cases, the energy of the normalized 3.5 sine wave (21.56) was 1.28 times bigger than for the normalized 2.5 sine wave (16.89).

**Figure 2:**
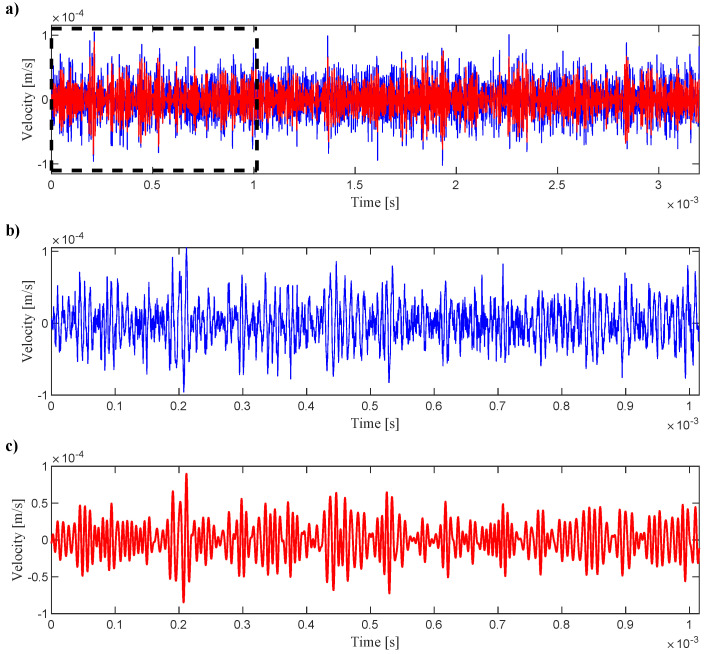
(**a**) The exemplary signal, measured with LDV, before (blue) and after (red) filtering. With the black dashed rectangle, the signal for further analysis is marked, and its zoom is shown in the diagram (**b**) before and (**c**) after filtering.

**Figure 3:**
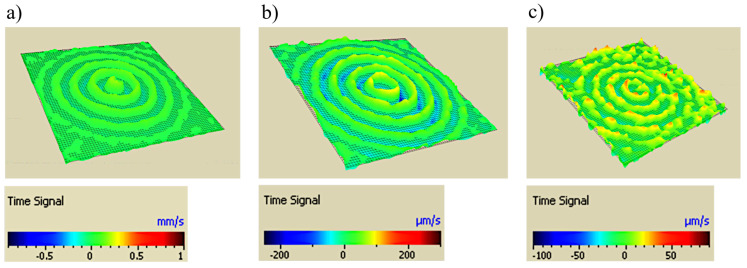
Examples of propagating waves for different gluing types: (**a**) WAX, (**b**) MAGIC, (**c**) Elastic glue. The time of measurement from the trigger: 0.2019 ms. Maps visualized by Politec software (Polytec Scanning Vibrometer, Version 9.0).

The sampling frequency was 2.56 MHz, and the length of the registered signal was 3.2 ms. After preliminary observation of the wave propagation, it was determined that in this task, the registered signal range should be narrowed down to 1 ms.The collected signals were filtered using a bandpass filter in the passband frequency 80–160 kHz and narrowed to the first 2600 elements (1 ms). An exemplary signal (for randomly selected point), measured with LDV before and after filtering and narrowing, is shown in Figure 2. In Figure 3, the maps of the neighborhood of the sensors are shown regarding the type of sensor gluing. Each map shows the same time step of 0.2019 ms after the trigger.

## 3. Calculation Procedure

**Figure 4:**
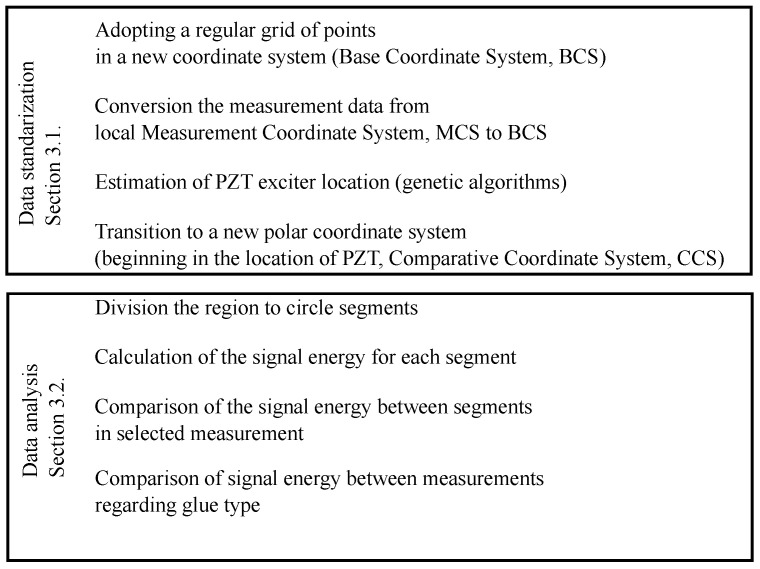
Scheme of the procedure.

The distance between the scanning head and the surface of the specimen was 230 cm. It is a relatively long distance, and even small changes in the position of the head influence the direction of the laser beam so that it does not reach the same point. The measurements lasted several weeks and required the equipment to be switched off between some of the samples. This is the first reason why the position of the scanning head may have changed. Another one is the necessity of changing the sample or measurement area. Due to technical reasons, it is impossible to obtain the identical location of measuring points (with such a dense mesh of points) concerning the sensor location. The new procedure presented here can help the comparison of on-the-surface propagated elastic waves.The algorithm of the procedure is shown in Figure 4.

### 3.1. Data Standardization

The location of measuring points during measurements by LDV is made in the local coordinate system of the tool (Measurement Coordinate System, MCS), which can be defined by the operator. It is possible to change the MCS every time while the observed area is changed, but in the author’s opinion, it is not necessary, as this process will not solve the problem of aligning the measuring points. Only the time consumption of the measurements is increased. The information of the distribution of the measurement points relative to each other is sufficient.

Assuming that the coordinates of the p-th point P in MCS are $P_{p}^{MCS}\left(x_{P}^{MCS},y_{P}^{MCS}\right)$, the new coordinate of the measuring points can be recalculated to a new Base Coordinate System (BCS) $P_{p}^{BCS}\left(x_{P}^{BCS},y_{P}^{BCS}\right)$ using the formula:

(1) $$\begin{array}{c} x_{P}^{BCS,1}=x_{P}^{MCS}-min\left(X^{MCS}\right)\\ y_{P}^{BCS,1}=y_{P}^{MCS}-min\left(Y^{MCS}\right)\\ x_{P}^{BCS}=x_{P}^{BCS,1}÷max\left(X^{BCS,1}\right)\;\cdot \;lx\\ y_{P}^{BCS}=y_{P}^{BCS,1}÷max\left(Y^{BCS,1}\right)\;\cdot \;ly \end{array}$$

where:

$X^{MCS}=\left[x_{1}^{MCS},x_{2}^{MCS},\ldots ,x_{p}^{MCS},\ldots ,x_{n}^{MCS}\right]$;
$Y^{MCS}=\left[y_{1}^{MCS},y_{2}^{MCS},\ldots ,y_{p}^{MCS},\ldots ,y_{n}^{MCS}\right]$;
n—number of measuring points;
$X^{BCS,1}=\left[x_{1}^{BCS,1},x_{2}^{BCS,1},\ldots ,x_{p}^{BCS,1},\ldots ,x_{n}^{BCS,1}\right]$;
$Y^{BCS,1}=\left[y_{1}^{BCS,1},y_{2}^{BCS,1},\ldots ,y_{p}^{BCS,1},\ldots ,y_{n}^{BCS,1}\right]$;
lx—the actual width of the area as measured on the object;
ly—the actual hight of the area as measured on the object.

The BCS is a Cartesian coordinate system, in which all measuring points are located in the first quadrant. Additionally, thanks to the normalization of coordinates and them referencing the real dimensions of the observed area (measured on the surface of the element), it is possible to avoid the influence of changes in the distance between the specimen and the scanning head on the location of the measuring points. Small differences in the distance are acceptable. This is especially important for large and heavy samples.

#### 3.1.1. Conversion of the Measurement Data to a New Grid of Points

**Figure 5:**
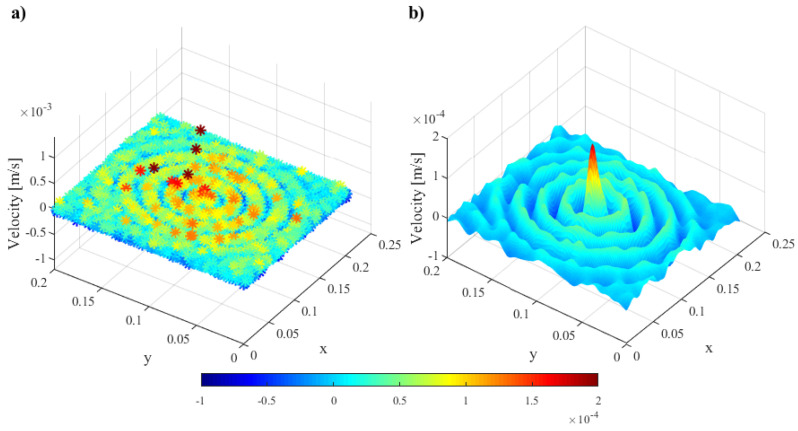
Exemplary view of (**a**) data obtained with LDV measurement, and (**b**) data after transformation to the BCS.

The data collected during LDV measurement are the time signal for each node of the measuring mesh. In Figure 5a, the exemplary velocities in the selected time step registered for all measuring points are shown. Due to specimen surface unevenness, the nodes of the mesh grid, despite assumptions, are not evenly spaced. Moreover, the measurement points that were located in places where the cables supplying the PZT transducers were located were not subject to measurements because the cables covered the surface of the element in which the wave propagated and the laser beam could not measure the wave. These factors made it even more difficult to compare the wave, induced by different sensors. To solve this problem, the conversion of the measurement data to a new regular grid of points was proposed as described below. The results of the data conversion are shown in Figure 5, where Figure 5a shows the data obtained with LDV measurement, while Figure 5b presents the results of data transformation to the BCS.

Starting from the beginning of the BCS, the regular grid of new nodes $N_{i,j}\left(x_{N_{i,j}},y_{N_{i,j}}\right)$ is determined, with a constant distance between rows ($d_{y}$) and columns ($d_{x}$). The number of rows *I* and columns *J* can be calculated as $I=l_{y}/d_{y}+1$, $J=l_{x}/d_{x}+1$, so i∈(1,2,…,I) and j∈(1,2,…,J). The number of nodes is nn=I·J, and does not have to be the same as the number of measuring points in MCS. For each node, the distance between the node and measuring points is calculated using Equation (2):

(2) $$r\left(N_{i,j},P_{p}\right)=\sqrt{{\left(x_{N_{i,j}}-x_{P}^{BCS}\right)}^{2}+{\left(y_{N_{i,j}}-y_{P}^{BCS}\right)}^{2}}$$

Next, a modified Hanning function [1] with the max value equal to 1 for the selected node and 0 for the points equally distant to $r_{max}$ (defined by the operator) is used as a weight function. See Equation (3):

(3) $$R\left(N_{i,j},P_{p}\right)=\left\{\begin{array}{cc} 0.5\;\cdot \;\left(1+cos\left(\frac{\pi \;\cdot \;r(N_{i,j},P_{p})}{r_{max}}\right)\right) & \mathrm{for}r\left(N_{i,j},P_{p}\right)<=r_{max}\\ 0 & \mathrm{for}r\left(N_{i,j},P_{p}\right)>r_{max} \end{array}\right.$$

Finally, the velocity for the selected node is calculated by weighted averaging Equation (4):

(4) $$AMPL\left(N_{i,j}\right)=\frac{\sum_{p=1}^{np}A(x_{P}^{BCS},y_{P}^{BCS})\;\cdot \;R\left(N_{i,j},P_{p}\right)}{\sum_{p=1}^{np}R\left(N_{i,j},P_{p}\right)}$$

where $A(x_{P}^{BCS},y_{P}^{BCS})$ is an amplitude of the signal registered with LDV for the p-th P point, and $AMPL(N_{i,j})$ is an amplitude of the signal calculated for the node $N_{i,j}$.

#### 3.1.2. Estimation of PZT Exciter Location

The wave propagation analysis should be made with consideration of the measuring point distance from the exciter. In view of the imprecise description of the location of the measurement grid nodes in relation to the sensor, and thus also the nodes of the new mesh, it became necessary to set the PZT location in another way. In this aim, genetic algorithms were used, assuming that the material was homogeneous and the elastic wave propagated in every direction in the same way.

For genetic algorithm implementation, the MATLAB environment was used. The ’ga’ function was used with default parameters (random initial population with a uniform distribution; population size = 50; the fraction of the population at the next generation = 0.8; stops criterion: the average relative change in the best fitness function value <= 1 × 10^−6^ and maximum number of iterations = 200). No linear or non-linear constraints were applied. Only a set of lower and upper bounds on the design variables (coordinates of the center of the circle) were limited to the dimensions of the analyzed area. The influence of parameter changes on the calculation result was not analyzed.

Based on 20 first time steps (before excitation), the noise level was estimated. The mean value $\bar{AMPL}$ and the standard deviation $\sigma_{AMPL}$ were established considering all nodes of the mesh grid. Then, for further analysis, another 41 time steps were selected. These were the steps in which the wider-and-wider propagated wave was observed. It was the first flight of the wave (without reflections from edges of specimen). As crucial points for the determination of the sensor location, the points satisfying Equation (5) were selected:

(5) $$B_{s}=\left\{N_{i,j}:AMPL_{s}\left(N_{i,j}\right)>=\bar{AMPL}+3\;\cdot \;\sigma_{AMPL}\right\}$$

where *s* is the time step number.

The coordinates of the circle center of gravity $\left[x_{c,s}^{BCS},y_{c,s}^{BCS}\right]$ were established for each time step using genetic algorithms, Equation (6):

(6) $$\left[x_{c,s}^{BCS},y_{c,s}^{BCS}\right]=ga\left(f_{o},R_{c},B_{s}\right)$$

where $f_{o}$ is the objective function defined in Equation (7), and

$R_{c}$—radius of the circle in the presented examinations, assuming that $R_{c}=70\mathrm{mm}$:

(7) $$f_{o}=\sum_{i}\sum_{j}{\left(R_{c}-\sqrt{{\left(x_{N_{i,j}}-x_{c,s}^{BCS}\right)}^{2}+{\left(y_{N_{i,j}}-y_{c,s}^{BCS}\right)}^{2}}\right)}^{2}\;\mathrm{for}N_{i,j}\in B$$

(8) $$x_{c}^{BCS}=\frac{\sum_{s}x_{c,s}^{BCS}}{S},\;y_{c}^{BCS}=\frac{\sum_{s}y_{c,s}^{BCS}}{S}$$

where *S* is the number of considered time steps.

**Figure 6:**
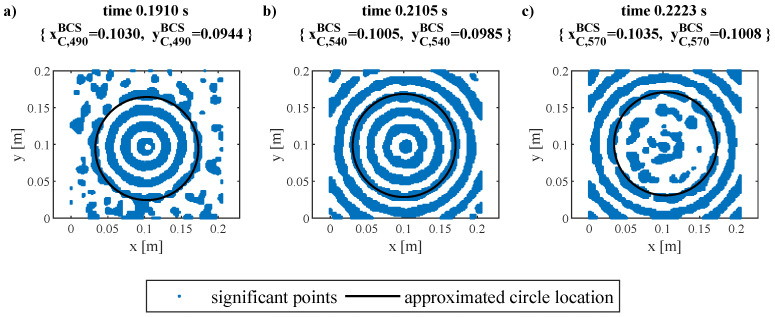
Significant points and GA-approximated locations of circle in (**a**) 490th, (**b**) 540th, (**c**) 590th time steps, for one selected exciter and its excitation.

The crucial points in the first, the middle, and the last time steps of exemplary considered sequence are shown in Figure 6. In these figures, also the location of circles approximated using the genetic algorithm are shown. The middle point of the circles corresponds to the PZT exciter location.

An example of the application of the proposed procedure is shown in Figure 6. The guided wave registered for all points of the grid in one selected moment in time can be visualized as a color map in 2D space. The applied color scale consists of two colors: blue for the points filling the Equation (5), and white for those with a smaller amplitude. The white ones are omitted in further analysis of the selected step. Each time step is taken into consideration by the genetics algorithm separately, so the obtained coordinates of the gravity center of the circle (shown with a black line) can be varied. It is the result of the nonideal character of the experimentally obtained data (noises, measurement uncertainties, imperfections of the specimen, etc., but primarily differences caused by the adhesion of the exciter). It is why more than one time step is used to determine the center location and justifies the use of Equation (8). The middle point of the circles corresponds to the PZT exciter location.

#### 3.1.3. Comparative Coordinate System (CCS)

The approximated location of PZT was established as an origin of the polar coordinate system, in which the coordinates of nodes were recalculated to the new ones, according to Equation (9): [23].

(9) $$\begin{array}{c} r_{N_{i,j}}^{CCS}=\sqrt{{\left(x_{N_{i,j}}^{BCS}-x_{c}^{BCS}\right)}^{2}+{\left(y_{N_{i,j}}^{BCS}-y_{c}^{BCS}\right)}^{2}}\\ \varphi_{N_{i,j}}^{CCS}=\left\{\begin{array}{cc} atan\left(\frac{y_{N_{i,j}}}{x_{N_{i,j}}}\right) & \mathrm{for}x_{N_{i,j}}>0,y_{N_{i,j}}>=0\\ atan\left(\frac{y_{N_{i,j}}}{x_{N_{i,j}}}\right)+2\pi & \mathrm{for}x_{N_{i,j}}>0,y_{N_{i,j}}<0\\ atan\left(\frac{y_{N_{i,j}}}{x_{N_{i,j}}}\right)+\pi & \mathrm{for}x_{N_{i,j}}<0\\ \frac{\pi }{2} & \mathrm{for}x_{N_{i,j}}=0,y_{N_{i,j}}>0\\ \frac{3\pi }{2} & \mathrm{for}x_{N_{i,j}}=0,y_{N_{i,j}}<0 \end{array}\right. \end{array}$$

### 3.2. Data Analysis

**Figure 7:**
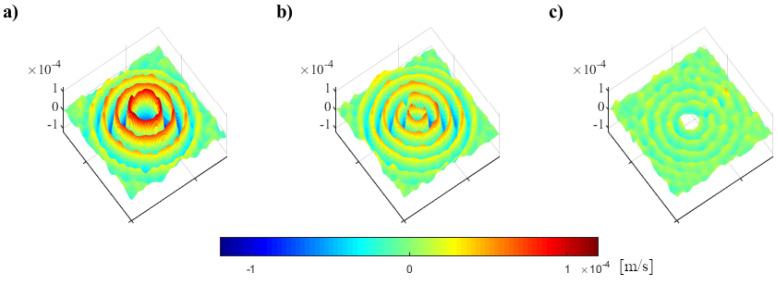
Examples of propagating waves for PZT sensors attached with: (**a**) WAX, (**b**) MAGIC, (**c**) Elastic glue, the same as in Figure 3 but after an application of the proposed procedure. The same scale was used for all maps in this figure.

Knowing the PZT sensor size, the nodes in the area of sensor should be omitted. They may cause interference in the data analysis (if some measurements were taken on the sensor surface, it is not the same surface on which the wave propagated in the analyzed material). Therefore, in further steps, the nodes with $r_{N_{i,j}}^{CCS}<=r_{sens}$ are not included. The adopted value of $r_{sens}$ equals 15 mm. Examples of propagating waves, the same as in Figure 3 but after an application of the proposed procedure, including the removal of nodes in the near neighborhood of the sensor, are shown in Figure 7.

**Figure 8:**
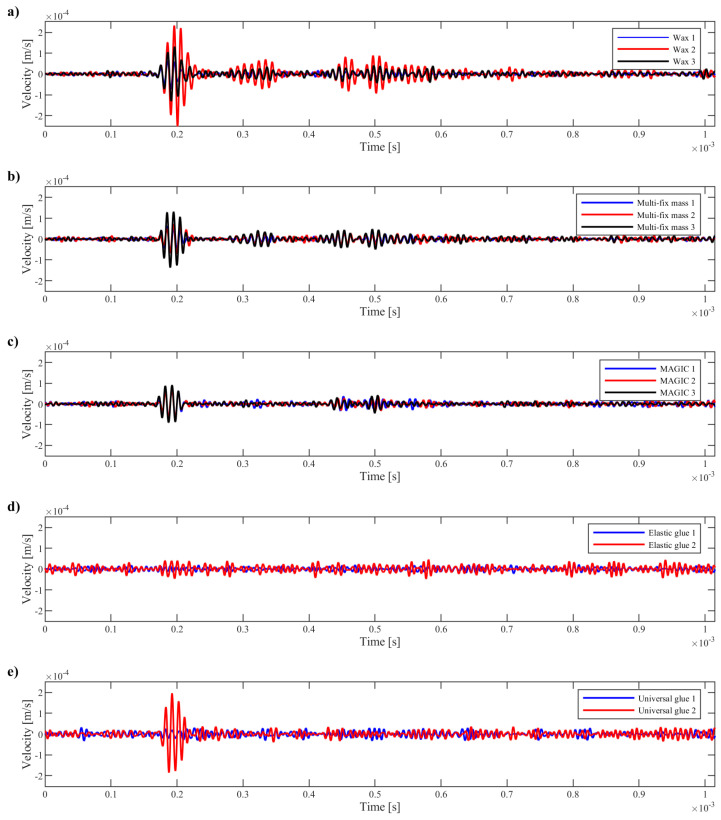
Exemplary comparison of signals collected during measurements with sensors glued on with different types of glue: (**a**) WAX, (**b**) Multi-fix mass, (**c**) MAGIC, (**d**) Elastic glue, (**e**) Universal glue. The comparison of nodes that were located closest to the origin of CCS (single selected node for each sensor).

The comparison of signals collected during measurements requires taking into account the location of the measuring node in relation to the excitation location. Thanks to the adoption of a new CCS, the selection of appropriate points is more objective. The exemplary signals, recorded for nodes located at the smallest distance from the origin of the polar system (CCS) and simultaneously with the smallest value of $\varphi_{N_{i,j}}$, are shown in Figure 8. The signals are grouped considering the type of applied glue.

As can be seen, despite the identical material properties and their homogeneity, as well as the same sensor sizes and excitation types, the registered signals may be completely different. It seems that the observed differences are the result of the way the sensor is glued. The applied glue influences the generated amplitude of the signal and also the possibility of sticking the sensor in an even and repeatable way, so the wave could propagate identically in every direction.

The highest amplitude of the propagated wave was obtained for wax, but as specified in Table 1, the sensor mounted on wax can be easily removed, e.g., by an unplanned jerk of the cable. In such a case, it is hardly possible to re-stick the sensor in the same way. In each of the measurements shown in Figure 8a, the shape of the registered signal is different. An even greater lack of repeatability of gluing is observed in the case of the use of elastic or universal glue. Despite no possibility of the accidental detachment of sensors, the application of this type of glue is not recommended due to the very low amplitude of the generated wave.

In the aim of the assessment of the generated wave quality, the circular area with $r_{circ}=80\;\mathrm{mm}$ and the middle point in the estimated location of the PZT sensor was adopted. The radius of this circle was established such that even in the case that the sensor was not in the center of the observed area, the circle would not be clipped. Then, this area was divided into twenty identical pieces, being slices of a circle (omitting the above-mentioned $N_{i,j}$, for which $r_{N_{i,j}}^{CCS}<=r_{sens}$).

The sum of the averaged squared amplitudes (ASAs) of the points, assumed to be significant, was calculated for each slice, considering the selected time steps. The calculations were made according to Equation (10):

(10) $$E_{a}=\sum_{s}\sum {\left\{AMPL_{s}\left(N_{i,j}\right)\right\}}^{2}\mathrm{for}N_{i,j}\in Aa$$

where a is the number of a piece, Aa is the area of the a-th piece, and s is the number of the selected time step s∈{490,492,494,…,570}.

**Figure 9:**
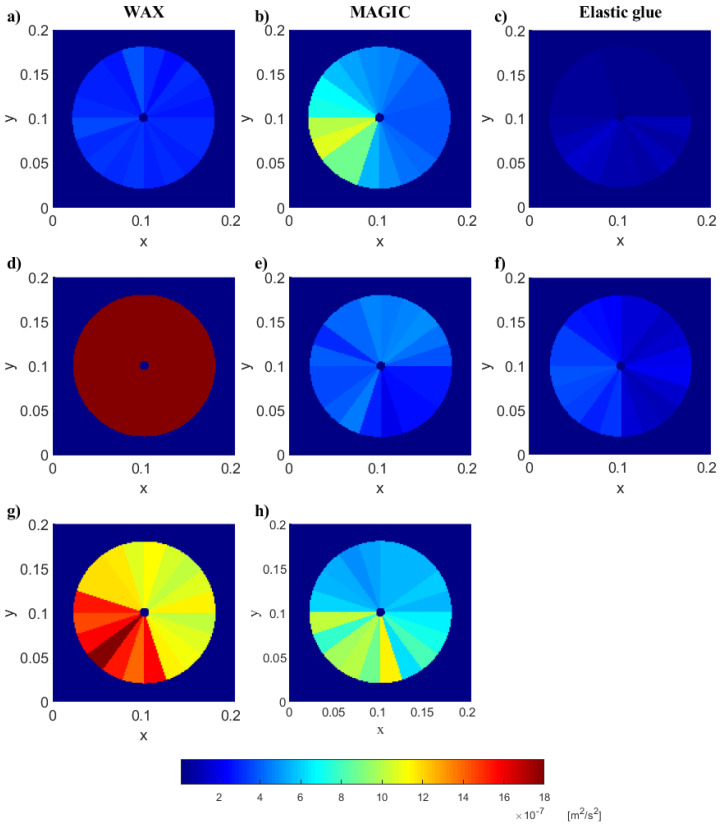
Averaged squared amplitudes of wave for selected pieces of the circular areas surrounding the exciters. The same scale was adopted to all shown examples. The excitation with 2.5 sine wave; the left column (**a**,**d**,**g**) sensors mounted with wax; the middle column (**b**,**e**,**h**)—commercial polyacetate-vinyl adhesive (glue MAGIC); the right column (**c**,**f**)—elastic ethyl 2-cyanoacrylate.

The exemplary comparison of changes in ASA for the selected pieces of the circular area is shown in Figure 9. The differences between the energy in the pieces are clearly visible. However, when choosing the type of sensor sticking, we should strive for relatively uniform excitation in all directions (as long as the tested material is homogeneous).

## 4. Results and Discussion

**Figure 10:**
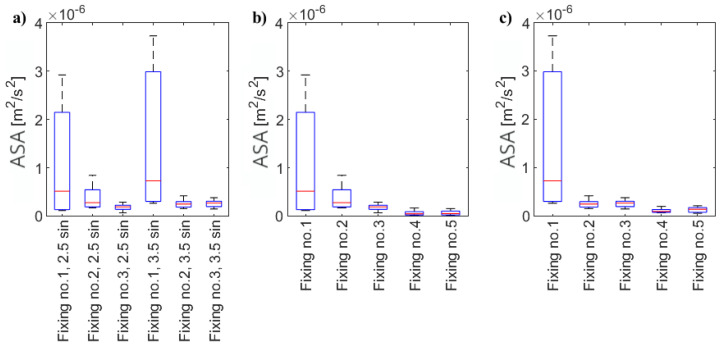
The comparison of ASA propagating in analyzed pieces of circular area. (**a**) Wave excited by one, the same, sensor NAC2013 regarding type of gluing and excitation type. (**b**) The comparison of ASA generated by different sensors. Excitation type: 2.5 sine wave. (**c**) The comparison of ASA generated by different sensors. Excitation type: 3.5 sine wave.

During the measurements, one (the same) sensor was used for all examinations with removable gluing (fixing type nos. 1–3). For each fixing and excitation type, three patterns were registered. The statistics of the collected results are shown in Figure 10. In the diagram, the energy of the wave propagating in selected pieces of a circular area is compared, considering the gluing type. As can be seen, for both types of excitation, the highest energy of the wave was obtained in the case of wax use. However, the variability of the analyzed energy is the biggest in this type of sticking. It means that the wave did not propagate evenly in all directions, and the comparison of the wave propagation between different points requires that this fact be taken into account. For both types of excitation, the most even wave propagation was obtained using commercial polyacetate-vinyl adhesive/glue MAGIC. The experiment was extended by gluing another four sensors but this time using two types of permanent glue (elastic and universal 2-cyanoacrylate). Four additional measurements with 2.5 sine wave excitation were collected, and the whole procedure was repeated. The results are shown in Figure 10b. Unfortunately, the observed wave amplitudes are significantly lower than in the previous cases, which result in a lower level of averaged squared amplitudes of waves. Additionally, as shown in Figure 8d,e, problems with the repeatability of the gluing are observed. The experiments were repeated for 3.5 sine wave excitation (Figure 10c). The trends obtained for the 2.5 sine wave excitation, which are described above, were confirmed.

## 5. Conclusions

In this article, two issues were raised, important from a practical point of view in elastic wave propagation measurements. The first one was the analysis of the influence of gluing the PZT actuators (applied glue types are listed in Table 1) to the examined surface on the elastic wave propagation. As shown, the highest amplitudes of waves, which correspond to the energy of the recorded signals, were obtained using wax, and the lowest ones using the permanent glue. Unluckily, the durability of the connections was increased with the decrease in the wave amplitudes. The stability of the sensors sticking is necessary; otherwise, they may accidentally be peeled off during the measurement. The paper shows that it is impossible to re-stick the sensor in the same way. The optimal solution in the analyzed task seems to be the application of bookbinding glue with a satisfactory amplitude of signal and durability of the connection. However, its application is narrowed only to rough, dry, and degreased surfaces.

The second issue is the development of the procedure dedicated to objective analysis and comparison of the elastic waves propagating on the surface of different specimens. In large-sized specimens, the precise location of the measurement points is impossible. Therefore, also a new procedure was developed, which engages genetic algorithms to help with the determination of a new coordinate system. In this system, wave propagation can be considered for the position of the exciter (not the measuring points). The proposed procedure also helps with the assessment of the quality of the wave propagation in different directions, and it is planned to be used in further examination of the analysis of the next flights of waves (e.g., after the reflection from the boundary of the material).

## Figures and Tables

**Table 1:** Applied fixing types.

Fixing Type No.	Glue	Advantages	Disadvantages
1	Wax	easy application	unstable (possible
		easy removal	accidental detachment)
			uniqueness of sticking
2	Multi-fix mass	easy application	long-drawn operation
		easy removal	of the sensor heats the mass
		more repeatable than wax	and changes its properties
3	commercial	easy application	poor bonding
	Polyacetate-vinyl	easy removal	on slippery surfaces
	adhesive∖	accidental	water-soluble
	glue MAGIC	detachment impossible	
		satisfactorily reproducible	
		gluing	
4	Elastic ethyl	easy application	low amplitude
	2-cyanoacrylate	accidental detachment	of generated signal
		impossible	very difficult to remove
			(detaching the sensor is
			associated with its damage)
5	Universal ethyl	easy application	low amplitude
	2-cyanoacrylate	accidental detachment	of generated signal
		impossible	very difficult to remove
			(detaching the sensor is
			associated with its damage)

## Data Availability

Date will be made available on reasonable request.
